# The gut–adipose/pancreas axis: a novel perspective on glycolipid metabolism dysregulation in MAFLD and T2DM pathogenesis

**DOI:** 10.3389/fendo.2025.1664233

**Published:** 2025-10-31

**Authors:** Jiahui Liang, Xingyu Chen, Yunhang Chu, Yan Leng

**Affiliations:** 1 College of Traditional Chinese Medicine, Changchun University of Traditional Chinese Medicine, Changchun, Jinlin, China; 2 Department of Liver, Spleen and Gastroenterology, Affiliated Hospital of Changchun University of Traditional Chinese Medicine, Changchun, Jilin, China

**Keywords:** metabolic-associated fatty liver disease, type 2 diabetes mellitus, gut microbiota, glucose and lipid metabolism, gut–adipose axis, gut–pancreatic axis

## Abstract

Metabolic-associated fatty liver disease (MAFLD) and type 2 diabetes mellitus (T2DM) frequently co-exist on the pathological basis of dysregulated glucose and lipid metabolism, forming a bidirectional causal relationship. The upstream mechanisms underlying this association require further elucidation. Recent studies suggest that the interactive network comprising the “gut–adipose axis” and “gut–pancreatic axis” represents a core component of the comorbidity mechanism. This network initiates with gut microbiota dysbiosis, which alters short-chain fatty acids (SCFAs), lipopolysaccharide (LPS), branched-chain amino acids (BCAAs), secondary bile acids(SBAs), and other microbial metabolites, as well as endocrine signals such as the endocannabinoid system (ECS) and incretin hormones. This network simultaneously influences adipose tissue and the pancreas to coordinate glucose and lipid homeostasis. Therefore, this paper proposes the “Common Messengers, Dual-Axis Convergence” model to systematically elucidate how the gut microbiota, through a shared set of messenger molecules, simultaneously and independently drives lipid and glucose metabolic dysregulation via the gut–adipose and gut–pancreatic axes, ultimately leading to the comorbidity of MAFLD and T2DM.

## 1 Introduction

In recent years, the network of interactions among the gut and other organs has become a research hotspot across different areas of physiology. These interactions exert extensive regulatory effects on host behavior, energy metabolism, appetite control, development, reproduction, and immune homeostasis. A growing body of research focuses on the regulation of glucose and lipid metabolism in the guy, aiming to explore new intervention targets (1, 2). Within this network, adipose tissue and the pancreas play pivotal roles in gut–organ crosstalk. Specifically, the “gut–adipose axis” and “gut–pancreas axis” establish bidirectional communication between the gut and distal metabolic tissues, thereby mediating the fine-tuned regulation of insulin resistance (IR), lipid homeostasis, and energy partitioning. This multilevel dialogue not only maintains glucose and lipid metabolic balance but also plays a key role in the pathological processes of both Metabolic-associated fatty liver disease(MAFLD) and type 2 diabetes mellitus (T2DM). The aim of this article is to reveal that the comorbidity of MAFLD and T2DM stems not only from the classic gut–liver axis cascade but also, more critically, from a “dual-axis branching” network originating from the gut as a common starting point. This means that gut dysbiosis generates a core set of messenger molecules. One branch of these messengers primarily drives lipid metabolic disorders via the “gut–adipose axis,” while the other branch primarily drives glucose metabolic disorders via the “gut–pancreatic islet axis.” These two signaling pathways act synergistically, jointly establishing the pathological foundation for comorbidity.

## 2 Strong association between MAFLD and T2DM under the influence of glucose and lipid metabolism

MAFLD is a metabolic disorder influenced by etiological factors such as abnormal lipid metabolism or IR. This disorder is characterized mainly by hepatic lipid droplet accumulation, hepatocyte steatosis, and histological abnormalities. MAFLD is currently among the most common chronic liver diseases. In fact, recent studies have indicated that its global prevalence has reached 39.22% (3, 4). The concept of MAFLD was proposed in 2020 by the Asia-Pacific Working Party on Non-Alcoholic Fatty Liver Disease (5). To highlight its alignment with complex systemic metabolic changes, the “multiple-hit pathogenesis” hypothesis (6–8) has been proposed, encompassing factors such as gut microbiota dysbiosis, IR, oxidative stress, inflammatory responses, hormonal imbalances, and dysglycaemia.

As the hepatic manifestation of metabolic disease, MAFLD often co-exists with other metabolism-related extrahepatic conditions. Cross-sectional data revealed a 65.33% global coprevalence of T2DM and MAFLD (9) and 96.82% of T2DM patients have metabolic dysfunction-associated steatohepatitis (MASH) (10). The co-morbidity of MAFLD and T2DM is believed to stem from disturbances in glucose and lipid metabolism. Furthermore (11), these two conditions may influence each other’s progression through direct or indirect pathways, ultimately forming the “triangular association” illustrated in **Table 1**.

**Table 1:** Core pathogenic mechanisms in the MAFLD and T2DM interplay: A vicious cycle model.

MAFLD	T2DM	Common pathological basis of interaction and vicious cycle	References	Cite
Abnormal Glucose Metabolism	Hepatic selective IR: Weakened insulin suppression of hepatic glucose output leads to fasting hyperglycemia. Consequence: Excessive glucose supply to the body.	Peripheral IR: Reduced glucose uptake and utilization by muscle and adipose tissues. Consequence: Results in persistent postprandial hyperglycemia.	Cycle 1: Increased hepatic glucose output in MAFLD → Worsens hyperglycemia in T2DM; Hyperglycemia and IR in T2DM → Promotes hepatic *de novo* lipogenesis, exacerbating MAFLD.	(113)
Abnormal Lipid Metabolism	Primary site: Liver. Characteristics: 1. ↑ Fatty acid synthesis: Via signaling pathways such as SREBP-1c. 2. ↓ Fatty acid oxidation and transport. 3. Consequence: Hepatic lipid accumulation, leading to lipotoxicity.	Primary site: Adipose tissue. Characteristics: 1. ↑ Lipolysis: Releases large amounts of FFA into the bloodstream. 2. Consequence: Elevated circulating FFA levels.	Cycle 2: Increased FFA release from adipose tissue in T2DM → FFA influx into the liver → Exacerbates hepatic lipid deposition in MAFLD → Hepatic lipotoxicity further impairs insulin signaling, aggravating systemic IR.	(114–116)

This table summarizes two core vicious cycles driving the co-progression of MAFLD and T2DM: The Hyperglycemia Cycle: MAFLD-driven increased hepatic glucose output worsens T2DM hyperglycemia, which in turn promotes hepatic lipogenesis. The Lipotoxicity Cycle: T2DM-enhanced adipose tissue lipolysis increases FFA flux to the liver, exacerbating hepatic steatosis and systemic IR. MAFLD, metabolic dysfunction-associated fatty liver disease; T2DM, type 2 diabetes mellitus; IR, insulin resistance; FFA, free fatty acids.

However, controversies remain regarding the specific mechanisms involved in this series of reactions, suggesting the existence of a common upstream pathway driving this process. Mounting evidence points to gut as the origin of this driving force.

## 3 The gut: the upstream hub of glucose and lipid metabolism abnormalities

Recent shifts in dietary patterns have propelled research on the gut and metabolic diseases into a new stage (12). Mechanistic studies have indicated that the gut influences energy metabolism homeostasis and glucose/lipid metabolism by regulating the gut microbiota, inflammatory responses, oxidative stress, and the neuroendocrine system (13). These mechanisms are closely related to the pathological progression of both MAFLD and T2DM. Consequently, the gut is regarded as the upstream hub of systemic metabolic disorders (14).

Daniel (15) proposed the hypothesis that disruption of the intestinal barrier and its associated dysbiosis could lead to IR and hepatic steatosis and accelerate the progression of metabolic syndrome, a view subsequently validated by further research. Metabolomic studies of body fluids (16) have revealed that distinct categories of metabolites are altered in metabolic disorders associated with gut microbiota dysbiosis. This dysbiosis is not a generalized change but manifests as a systematic imbalance of specific bacterial taxa with crucial metabolic functions, directly regulating the levels and activity of messenger molecules.

This is primarily reflected in a reduction in protective bacteria: core probiotics such as *Akkermansia muciniphila* and *Faecalibacterium prausnitzii* (17, 18) which enhance the gut barrier and ferment dietary fibers to produce SCFAs, are often significantly reduced in patients with MAFLD and T2DM. Their depletion directly leads to insufficient SCFAs production, thereby weakening their multiple protective effects, including nourishing intestinal epithelial cells, exerting anti-inflammatory effects, and stimulating glucagon-like peptide-1(GLP-1) secretion. Another key aspect is the expansion of conditional pathogens: some LPS-rich gram-negative bacteria, such as *Escherichia coli* (19) from the phylum Proteobacteria, may overgrow. The increase in such bacteria serves as an intrinsic source for persistently elevated circulating LPS levels, providing a continuous trigger for driving chronic inflammation and IR via the TLR4/NF-κB pathway.

Furthermore, the imbalance of metabolically functional bacteria is equally critical: an abnormal abundance of bacteria responsible for converting primary to SBAs (secondary bile acids), such as *Clostridium scindens* (20), directly disrupts the composition and dynamics of the entire bile acid pool. This not only affects lipid digestion but also, more importantly, interferes with the normal activation of metabolic receptors such as FXR and TGR5 by SBAs, thereby disrupting glucose and energy metabolism homeostasis (**Table 2**). Additionally, at the mechanistic level, studies further indicate that bacteria such as *Prevotella* and certain Bacteroides can participate in the regulation of IR by influencing imidazole propionate concentrations (21–25).

**Table 2:** Metabolites produced by probiotics alone or through interactions with the gut microbiota and their effects.

Source	Related products & derivatives	Source & affected microbiota	Effects	Mechanism	Refs.
Dietary Fiber	SCFA: butyrate	*Firmicutes*	Maintains mucosal integrity, regulates local and systemic immunity, reduces obesity, stimulates leptin synthesis, releases anorexigenic hormones.	The three SCFAs modulate the expression of PPAR-γ, GPR43 or FFAR2, GPR41 (or FFAR3), and GPR109A (butyrate only). Acetate influences the intestinal IgA antibody response. Butyrate: inhibits HDAC activity and suppresses prototypical pro-inflammatory signaling pathways (such as the Nuclear Factor kappa B, or NF-κB, pathway).	(117–124)
	SCFA: propionate	*Bacteroidetes*	Reduces weight gain, intestinal and hepatic gluconeogenesis, and levels of pro-inflammatory cytokines.		
	SCFA: acetate	*Blautia*, *Bifidobacterium*	Increases satiety, reduces body weight, improves insulin sensitivity, and decreases pro-inflammatory cytokines.		
Saturated Fat	LPS	Produced by gram-negative bacteria, e.g., *Enterobacteriaceae*	Associated with IR, visceral fat accumulation, and increased intestinal permeability.	Activates Cells expressing TLR-4, increasing inflammation in WAT.	(125)
Primary Bile Acids	SBAs	*Firmicutes* (e.g., *Clostridium* clusters)	In excess: associated with intestinal inflammation, DNA damage, increased cancer risk. In appropriate amounts: participates in host metabolism regulation.	Act as signaling molecules activating the FXR and TGR5, thereby regulating glucose, lipid metabolism, and energy expenditure. In excess, exhibit cytotoxic and pro-inflammatory effects.	(126, 127)
Protein	BCAAs	*Bacteroides*, *Prevotella*	High levels are associated with an increased risk of IR and T2DM.	Likely interferes with insulin signal transduction by activating the mTOR and p70S6K signaling pathways, leading to IR.	(128)
	Ammonia, hydrogen sulfide, phenols, etc.	*Bacteroides*, *Clostridium*, *Prevotella*, etc.	Cytotoxic at high concentrations, damages intestinal epithelial cells, increases intestinal permeability, promotes inflammation.	Induces oxidative stress and activates pro-inflammatory signaling pathways (e.g., the NF-κB pathway).	(129, 130)
	N-oxide (TMAO)	*Firmicutes* (e.g., *Clostridium*), *Proteobacteria*	Dose-dependent dual effects.	At high concentrations (e.g., 80 μM), TMAO impairs β-cell mitochondrial function and induce endoplasmic reticulum stress, thereby inhibiting insulin secretion; at lower concentrations (e.g., 40 μM), it may act as a chemical chaperone, alleviating β-cell apoptosis under glucolipotoxicity.	(131)

BCAAs, branched-chain amino acids; ER, endoplasmic reticulum; FFAR, free fatty acid receptor; FXR, farnesoid X receptor; GPR, G-protein-coupled receptor; SBAs, secondary bile acids; HDAC, histone deacetylase; IgA, immunoglobulin A; IR, insulin resistance; LPS, lipopolysaccharide; mTOR, mechanistic target of rapamycin; NF-κB, nuclear factor kappa B; PPAR-γ, peroxisome proliferator-activated receptor gamma; SCFA, short-chain fatty acid; T2DM, type 2 diabetes mellitus; TLR4, Toll-like receptor 4; TMAO, trimethylamine N-oxide; WAT, white adipose tissue.

**Figure 1:**
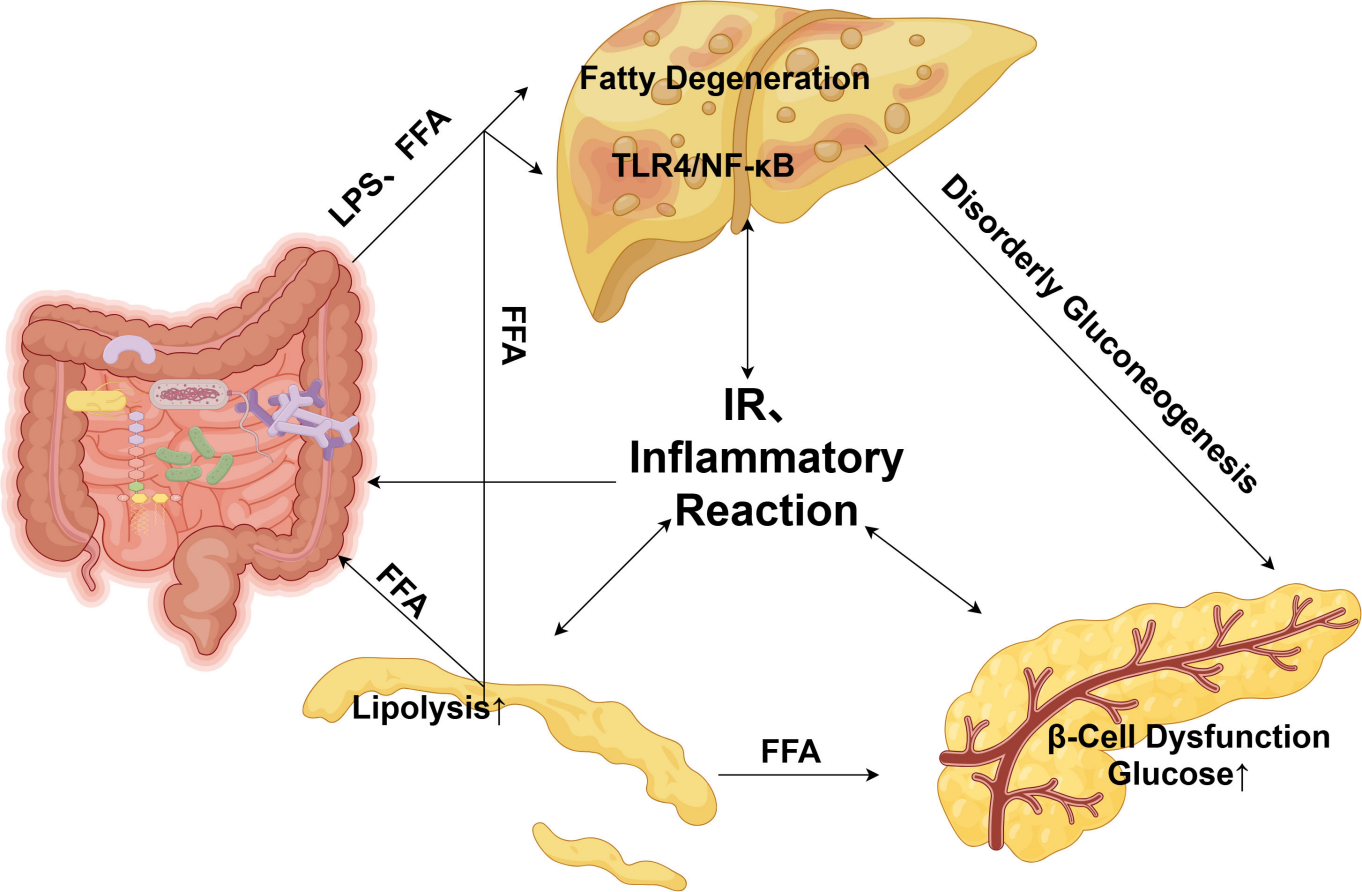
The gut–adipose–pancreas crosstalk cascade. (The schematic illustrates a self-perpetuating vicious cycle. Solid arrows indicate direct promoting actions or metabolic fluxes, while dashed arrows represent reinforcing feedback loops. Key pathophysiological steps include: 1) Gut dysbiosis and increased intestinal permeability; 2) Hepatic influx of bacterial LPS and FFAs via the portal vein; 3) Activation of pro-inflammatory pathways (e.g., TLR4/NF-κB) and induction of HIR; 4) Compensatory pancreatic β-cell hypersecretion progressing to exhaustion under glucolipotoxicity; 5) Systemic IR promoting adipose tissue lipolysis; and 6) Release of inflammatory factors from liver and adipose tissue, fueling systemic inflammation. This interconnected network, centered on IR, collectively propels disease progression from steatosis to steatohepatitis and overt diabetes. LPS, lipopolysaccharide; FFAs, free fatty acids; TLR4, Toll-like receptor 4; NF-κB, nuclear factor kappa B; HIR, hepatic insulin resistance; IR, insulin resistance.).

The dysbiosis-driven disruption of various metabolites not only establishes the molecular basis for MAFLD and T2DM comorbidity but also triggers a cascade of metabolic abnormalities. This cascade, depicted in **Figure 1**, originates in the gut and extends through a network of key organs, including the liver, pancreas, and adipose tissue.

Initiated by dysbiosis, harmful gut-derived products including LPS and FFAs translocate into the liver via the portal circulation. Within the liver, such substances trigger lipid deposition and activate inflammatory pathways like TLR4/NF-κB, leading to hepatic insulin resistance and MASH development. Concurrently, pancreatic β-cells compensate through insulin hypersecretion, causing hyperinsulinaemia. Combined with HIR-driven uncontrolled gluconeogenesis, a state of persistent hyperglycaemia emerges. Chronic glucolipotoxicity then promotes β-cell functional exhaustion and overt T2DM onset (26, 27). The disorder amplifies systemically as insulin-resistant adipose tissue increases lipolysis, flooding the liver with additional FFAs and further exacerbating hepatic steatosis and inflammation. Ultimately, compromised liver and adipose tissues secrete copious inflammatory factors, generating a systemic inflammatory state which intensifies insulin resistance and β-cell dysfunction across all tissues. A self-perpetuating, vicious cycle results, centered on the “gut-liver-pancreas-adipose tissue” axis, interconnected by worsening propelling disease progression (28).

Throughout this process, hormones, inflammatory factors, and metabolites interact through a complex network, leading to a cascading failure of the metabolic network and jointly driving metabolic imbalance into a cyclical cascade reaction of “glucose and lipid accumulation → metabolic dysregulation → organ injury” (29, 30).

However, this classical cascade view is insufficient to fully explain the high frequency of MAFLD and T2DM comorbidity. Unger et al. introduced the concept of the “gut–pancreas axis” in 1969 (31); and scholars have subsequently proposed the “gut–adipose axis” in recent years (32). Building on these advances, we recognize that the influence of the gut on comorbidities involves a parallel and crucial core mechanism beyond the classic cascade of the gut–liver–pancreas–adipose axis.

Therefore, we have distilled the “Common Messengers, Dual-Axis Branching” model of MAFLD and T2DM comorbidity. The core premise of this model is that gut microbiota dysbiosis triggers not an isolated change in a single molecule but rather a collective dysregulation of a core set of messenger molecules. These “common messengers” are simultaneously and precisely directed via two relatively independent communication pathways—the “gut-adipose axis” and the “gut-pancreatic islet axis”—to targets beyond the reach of the aforementioned cascade reaction. Specifically, they directly disrupt lipid metabolic homeostasis in adipose tissue and glucose metabolic regulation in the pancreas, respectively. In this process, the gut acts as the source, while adipose tissue and the pancreas serve as the key effector organs.

## 4 Key messengers linking the gut–adipose axis and the gut–pancreatic islet axis

In this model, the “gut-pancreatic axis (33, 34)” is a bidirectional pathway between the intestine and pancreatic islets that coordinates insulin secretion, glucose homeostasis, and β-cell protection. It integrates neuroendocrine signals (e.g., vagal reflexes, GLP-1/GIP hormones), immune cues (e.g., cytokines), and microbial metabolites (e.g., SCFAs) to regulate pancreatic function. These mechanisms collectively enhance insulin release, suppress glucagon, mitigate inflammation, and maintain β-cell integrity, ensuring precise systemic glucose control.

The “gut-adipose axis (35, 36)” refers to a bidirectional communication network between the gut and adipose tissue. It finely regulates systemic lipid storage, breakdown, and energy expenditure through the following key messenger mechanisms: (1) gut hormones (e.g., GLP-1, PYY) directly regulate adipocyte differentiation and lipolysis; (2) sympathetic nerve activation promotes lipolysis; (3) microbial metabolites (e.g., SBAs) modulate systemic energy expenditure and lipid metabolism by activating host receptors such as FXR and TGR5; and (4) inflammatory responses, among others. By coordinating intestinal nutrient absorption and adipokine secretion, these integrated mechanisms collectively maintain adipose tissue homeostasis.

We propose that the co-morbidity of MAFLD and T2DM can be traced to a core set of gut-derived signaling molecules. These messengers diverge to engage the “gut-adipose axis” and the “gut-pancreatic axis”, perturbing the metabolic homeostasis of adipose tissue and the pancreas, respectively. The ensuing organ-specific dysfunctions then converge to establish a systemic comorbid network. Subsequent sections will delineate how specific messengers—such as SCFAs, LPS, BCAAs, and SBAs—orchestrate these divergent effects within the two axes.

### 4.1 Microbial metabolites: key pathways regulating glucose and lipid metabolism

#### 4.1.1 Lipopolysaccharide

##### 4.1.1.1 Effects on the gut–pancreatic islet axis

When gut microbiota dysbiosis and impaired barrier function occur, LPS derived from gram-negative bacteria is translocated into the systemic circulation, primarily affecting glucose metabolism by inducing chronic low-grade inflammation (37). It disrupts pancreatic endocrine function and glucose homeostasis through three mechanisms: “inflammatory toxicity,” “signal interference,” and “hormone suppression.”

*In vivo* experiments in mice have demonstrated that LPS activates immune cells via the TLR4/NF-κB pathway, leading to the production of pro-inflammatory cytokines (e.g., TNF-α, IL-1β) and the induction of inducible nitric oxide synthase (iNOS). iNOS catalyzes the massive production of nitric oxide (•NO), which rapidly reacts with superoxide (O_2_•^−^) to form potent biological oxidants such as peroxynitrite (ONOO^−^). Peroxynitrite directly damages mitochondrial function in β-cells, leading to apoptosis (38). The aforementioned inflammatory cytokines can activate kinases such as JNK and IKKβ, interfere with insulin receptor substrate-1 (IRS-1) function, and downregulate GLUT2 expression, thereby impeding insulin signal transduction and reducing insulin sensitivity (39, 40). Furthermore, LPS can modulate glucose homeostasis by affecting the LPS/GLP-1 pathway. Another animal study demonstrated that LPS reduces L-cell viability, increases TNF-α levels, induces apoptosis, and decreases both the mRNA and protein levels of GLP-1, hindering insulin release (41, 42).

##### 4.1.1.2 Effects on the gut–adipose axis

LPS enters the circulation via two principal routes: the paracellular pathway under impaired tight junctions, and the transcellular pathway during fat absorption after a high-fat diet. The latter utilizes lipid rafts and CD36, allowing LPS to bind to chylomicrons and access the lympho−hematogenous circulation (43), thereby promoting fat storage, accelerating lipolysis, and worsening lipotoxicity.

LPS activates the TLR4/NF-κB pathway in adipose tissue, inducing the expression of factors such as TNF-α. These factors, in turn, inhibit IRS-1 via JNK/IKKβ signaling, block the antilipolytic effect of insulin, and promote the phosphorylation of hormone-sensitive lipase (HSL), accelerating triglyceride (TG) hydrolysis and FFA release (44–46). Additionally, LPS can stimulate the sympathetic nervous system, increasing catecholamine release, which activates the cAMP/PKA pathway via β-adrenergic receptors (47), further promoting HSL phosphorylation and lipolysis. Additionally, LPS inhibits PPARγ activity in adipocytes within mice, reducing adiponectin secretion, which further exacerbates lipolysis and IR (48). Intestinal LPS can also activate the TLR/MyD88 signaling axis, upregulating the expression of Rev-Erbα in small intestinal epithelial cells, directly increasing dietary lipid absorption and exacerbating the lipid load from the source (49). Consequently, LPS promotes adipose tissue dysfunction, elevated systemic FFA levels, and ectopic lipid deposition in the liver through both direct and indirect pathways.

Comorbidity mechanism: LPS translocation under gut microbiota dysbiosis simultaneously activates inflammation in both adipose and pancreatic tissues. On the one hand, it impairs β-cell function, suppresses GLP-1, and exacerbates peripheral IR; on the other hand, it promotes adipose tissue inflammation, lipolysis, and lipid absorption. These two major pathways collectively lead to the deterioration of the “glucolipotoxic” environment, ultimately synergistically driving intrahepatic lipid deposition in MAFLD and the loss of glycaemic control in T2DM, thereby establishing comorbidity.

#### 4.1.2 Short-chain fatty acids

SCFAs (50), primarily comprising acetate, propionate, and butyrate, are key metabolites produced by the gut microbiota through the fermentation of dietary fiber. They play a central role in maintaining glucose and lipid metabolic homeostasis.

##### 4.1.2.1 Effects on the gut–pancreatic islet axis

SCFAs preserve pancreatic function and glucose homeostasis via multiple mechanisms, particularly butyrate. First, butyrate and others activate the GPR43 receptor on intestinal L cells, stimulating the release of GLP-1, thereby enhancing glucose-dependent insulin secretion in mice (51). Second, *in vivo* animal studies have demonstrated that SCFAs, acting as a histone deacetylase (HDAC) inhibitor, increases histone acetylation levels to promote the expression of anti-inflammatory genes such as the NF-κB inhibitor protein (IκBα). This subsequently inhibits NF-κB signaling and reduces the production of inflammatory cytokines such as IL-1β. Concurrently, SCFAs can competitively inhibit the binding of LPS to TLR4, synergistically decreasing the synthesis of TNF-α and IL-6, thereby alleviating local islet inflammation and protecting mice β-cells from inflammation-induced apoptosis (52–55). Furthermore, butyrate activates the nuclear factor erythroid 2-related factor 2 (Nrf2) pathway, promoting its nuclear translocation and upregulating the expression of antioxidant enzymes such as superoxide dismutase (SOD) and glutathione peroxidase (GPx), effectively mitigating oxidative stress and mitochondrial damage in mice β-cells (56).

##### 4.1.2.2 Effects on the gut–adipose axis

Regarding lipid metabolism, SCFAs alleviate lipotoxicity by coordinating the functions of adipose tissue and the liver. An *in vivo* animal experiment has confirmed that, in adipose tissue, SCFAs activate GPR43 and GPR41 receptors. Through pathways involving PPARγ, they upregulate lipogenesis and promote fatty acid oxidation, ultimately inhibiting lipolysis, promoting normal adipocyte differentiation, helping to reduce circulating FFA levels, and improving ectopic lipid deposition and insulin sensitivity (57, 58). Butyrate can also serve as a preferential energy substrate, promoting the expression of peroxisome proliferator-activated receptor gamma coactivator 1-alpha (PGC-1α) and mitochondrial uncoupling protein 1 (UCP1) in brown adipose tissue, thereby accelerating fat metabolism and thermogenesis (59). Their immunomodulatory role is equally crucial: by inhibiting TLR4/NF-κB signaling, SCFAs drive the polarization of adipose tissue macrophages from the pro-inflammatory M1 phenotype to the anti-inflammatory M2 phenotype, thus improving IR and indirectly regulating fat accumulation (60). Additionally, upon reaching the liver, propionate can directly inhibit the expression of fatty acid synthase (FAS) and sterol regulatory element-binding protein 1c (SREBP-1c), effectively reducing *de novo* lipogenesis in the liver and intervening at the source in the progression of MAFLD (61).

Comorbidity mechanism: A reduction in the abundance of SCFAs weakens their dual protective effects on both the gut-pancreatic islet axis and the gut-adipose axis. In the pancreas, diminished stimulation of GLP-1, anti-inflammatory activity, and antioxidant activity collectively exacerbate β-cell dysfunction and insulin resistance. In adipose tissue, a decrease in the ability to promote healthy fat storage, thermogenesis, and anti-inflammatory effects leads to increased lipolysis, ectopic deposition, and hepatic *de novo* lipogenesis. This collective loss of protective signaling disrupts the balance of glucose and lipid metabolism, ultimately synergistically driving the comorbid progression of MAFLD and T2DM.

#### 4.1.3 Branched-chain amino acids

Blood concentrations of BCAAs, which are essential amino acids, are influenced not only by dietary intake but also by the metabolic activity of the gut microbiota (e.g., *Prevotella* and *Bacteroides*). Gut dysbiosis leads to reduced BCAAs breakdown, causing abnormally elevated circulating BCAAs levels. This exerts profound effects on glucose and lipid metabolism through excessive activation of mechanistic target of rapamycin complex 1 (mTORC1) signaling.

##### 4.1.3.1 Effects on the gut–pancreatic islet axis

Elevated BCAAs levels have multiple adverse effects on pancreatic function. In pancreatic β cells, chronic high concentrations of BCAAs induce sustained activation of mTORC1 signaling. On the one hand, this inhibits the key autophagy kinase ULK1, impairing the clearance of damaged mitochondria and leading to mitophagy defects and dysfunction. On the other hand, it causes excessive protein accumulation in the endoplasmic reticulum, inducing endoplasmic reticulum stress (ERS). Together, these ultimately lead to β-apoptosis and inhibit insulin synthesis and secretion (62, 63). Extensive *in vitro* and *in vivo* studies have demonstrated that in peripheral tissues, excessive mTORC1 activation inhibits PI3K/Akt insulin signaling pathway transduction, reduces Akt phosphorylation, and consequently inhibits the Rab protein-mediated translocation of GLUT4 to the cell membrane, reducing glucose uptake and directly causing IR (64–66).

##### 4.1.3.2 Effects on the gut–adipose axis

BCAAs alter fat metabolism in a way that promotes storage and inhibits expenditure. After entering adipocytes, the catabolic product of BCAAs, Ac-CoA, can acetylate the brown fat transcription factor PRDM16, enhancing its binding to PPARγ. This process promotes white adipogenesis while inhibiting the transformation of white to brown/beige fat, thereby reducing energy expenditure and promoting lipid droplet formation and fat accumulation (67). Furthermore, Furthermore, extensive *in vivo* animal studies demonstrate that leucine can bind to and relieve the inhibition of the mTORC1 inhibitory protein Sestrin2, thereby activating SREBP-1c. This promotes the conversion of acetyl-CoA to FFAs and the subsequent esterification of these fatty acids into TG, resulting in the formation of lipid deposits in adipocytes and hepatocytes and directly exacerbating the pathological process of MAFLD (68–70).

Comorbidity mechanism: Abnormally elevated circulating BCAAs levels constitute a common pathological signal linking metabolic disorders in the pancreas and adipose tissue. In the pancreas, they impair β-cell function and survival via mTORC1 and exacerbate peripheral insulin resistance. In adipose tissue, they alter the metabolic program of fat cells, promoting lipid storage and inhibiting energy expenditure. This synchronous occurrence of “impaired islet function” and “lipid metabolism shifted toward storage” provides the core driving force for intrahepatic lipid deposition in MAFLD and loss of glycaemic control in T2DM, forming a unique comorbidity pathway.

#### 4.1.4 Secondary bile acids

SBAs are key signaling molecules produced by the gut microbiota (e.g., Clostridium species) from primary bile acids. They primarily regulate the host’s glucose and lipid metabolic balance by activating FXR and TGR5. *In vivo* studies have shown that disrupting the gut microbiota perturbs bile acid metabolism, inhibits FXR signaling, and consequently triggers glucose and lipid metabolic disorders, whereas supplementation with FXR agonists can significantly ameliorate these abnormalities (71).

##### 4.1.4.1 Effects on the gut–pancreatic islet axis

SBAs exert important regulatory effects on pancreatic function and glucose homeostasis through receptor signaling, as demonstrated in *in vivo* animal studies. First, TGR5 receptor activation upregulates the expression of IκBα, anchoring NF-κB in the cytoplasm and preventing its nuclear translocation, thereby inhibiting the transcription of pro-inflammatory genes. This anti-inflammatory mechanism helps protect β cells from inflammatory damage (72). More importantly, SBAs can improve glucose homeostasis by activating the intestinal FXR/TGR5-GLP-1 axis, stimulating intestinal L cells to secrete GLP-1, which subsequently promotes the closure of ATP-sensitive potassium channels in pancreatic β cells, leading to membrane depolarization and calcium influx and ultimately enhancing glucose-stimulated insulin secretion (73).

##### 4.1.4.2 Effects on the gut–adipose axis

In terms of lipid metabolism, SBAs co-ordinately regulate lipid storage and consumption through FXR in different organs. An *in vivo* animal study revealed that in adipose tissue, SBAs activate FXR and, through pathways such as upregulating ApoC2, significantly increase the expression of mitochondrial UCP1 and PGC1α in beige adipocytes, promoting the browning of WAT and thereby increasing energy expenditure (74). In the liver, FXR activation upregulates PPARα, enhances mitochondrial β-oxidation capacity in hepatocytes, accelerates FFA clearance, and reduces the hepatic lipid burden (75, 76). Furthermore, the activation of intestinal FXR can directly regulate the intestinal absorption of lipids, modulating lipid metabolism from the source and showing great potential for preventing and treating MAFLD (77, 78).

Comorbidity mechanism: Dysregulation of secondary bile acid metabolism essentially represents the disruption of an important signaling network connecting the gut, pancreas, and adipose tissue. When their production is abnormal, the protective effects on the gut-pancreatic islet axis are weakened, exacerbating hyperglycaemia and β-cell damage. Moreover, the ability to promote adipose thermogenesis and enhance hepatic lipolysis via the gut–adipose axis is also reduced, leading to ectopic lipid deposition. Therefore, the imbalance in the secondary bile acid signaling network simultaneously impairs the fine regulation of both glucose metabolism and lipid metabolism, promoting the synergistic progression of MAFLD and T2DM.

### 4.2 Hormones: effectors regulating glucose and lipid metabolism

#### 4.2.1 Endocannabinoid system

The endocannabinoid system (ECS) is a complex signaling network composed of endocannabinoids, their synthesis and degradation enzymes, and cannabinoid receptors (e.g., CB1R and CB2R). It is widely distributed in the brain, gastrointestinal tract, liver, adipose tissue, pancreas, and other tissues and is among the core systems that regulate energy balance, glucose/lipid metabolism, and immune inflammation (79). Gut microbiota dysbiosis can alter ECS tone, whereas dietary interventions and probiotics can modulate receptor expression, thereby improving metabolic syndrome (80).

##### 4.2.1.1 Effects on the gut–pancreatic islet axis

The ECS bidirectionally regulates pancreatic secretory function, with the effect depending on the activated receptor subtype. CB2R activation has protective effects on glucose metabolism; in pancreatic β-cells, CB2R activation enhances glucose-induced Ca^2+^ oscillations, thereby promoting insulin secretion (81). Furthermore, in immune cells (e.g., ILC2s), CB2R agonism can improve established IR and restore glucose tolerance by activating the AKT, ERK1/2, and CREB pathways (82). In contrast, excessive activation of CB1R is typically associated with metabolic deterioration.

##### 4.2.1.2 Effects on the gut–adipose axis

The ECS, particularly CB1R, plays a key role in regulating lipid metabolism in adipose tissue and the liver. CB1R activation is a core pathway that promotes lipid storage and exacerbates MAFLD; experiments conducted at both the cellular and animal levels have demonstrated that, in adipose tissue, CB1R activation stimulates p38 MAPK phosphorylation, reduces adiponectin production, and inhibits the expression of the thermogenic gene UCP1, thereby reducing energy expenditure and promoting fat accumulation (83, 84). In the liver, CB1R activation promotes SREBP-1c expression, driving *de novo* lipogenesis (DNL), while simultaneously inhibiting fatty acid β-oxidation and reducing FFA clearance, directly leading to intrahepatic lipid deposition (85, 86). Therefore, CB1R inhibitors such as rimonabant can effectively improve systemic lipid metabolic homeostasis by reversing these processes.

Comorbidity mechanism: An imbalance in ECS tone is an important bridge connecting pancreatic dysfunction with adipose/liver metabolic disorders. When CB1R signaling is overactive but protective CB2R signaling is insufficient, it simultaneously leads to the following: in the pancreas, dysregulated insulin secretion control and exacerbated insulin resistance; in adipose tissue and the liver, increased lipid storage, decreased energy expenditure, and enhanced hepatic *de novo* lipogenesis. This synergistic action of signals promoting lipid deposition and impairing glucose-lowering function drives the comorbidity of MAFLD and T2DM, making the ECS a highly promising therapeutic target.

#### 4.2.2 Incretins

Incretins, primarily GLP-1 and GIP, are core hormones secreted by the gut in response to nutrient intake. They play a pivotal role in communication between the gut and distant metabolic organs.

##### 4.2.2.1 Glucose-dependent insulinotropic polypeptide

GIP is secreted by K cells in the proximal small intestine, and its release is triggered primarily by glucose and lipids. With respect to the gut–pancreatic islet axis, GIP binds to the GIPR on β cells, activating the cAMP-PKA/EPAC2 signaling pathway and significantly enhancing glucose-stimulated insulin secretion. Furthermore, a study combining *in vitro* cellular models with human and mice pancreatic islet tissue confirmed that under hypoglycaemic conditions, GIP can act on pancreatic α cells to stimulate glucagon release and, via activation of the ERK/CREB pathway, exert protective and regenerative effects on β cells similar to the effects of GLP-1, collectively maintaining glucose homeostasis (87–89).

Regarding the gut–adipose axis, GIP activates GIPR in adipocytes and the cAMP/PKA/CREB pathway, upregulating the expression and activity of lipoprotein lipase (LPL). This promotes the storage of TGs in adipose tissue, reduces ectopic lipid deposition at sites such as the liver and muscle, and helps alleviate the severity of MAFLD (90–93).

##### 4.2.2.2 Glucagon-like peptide-1

GLP-1 is secreted by L cells in the distal ileum and colon. Its role in the gut–pancreatic islet axis, as mentioned earlier, centers on its potent stimulation of insulin secretion. Its effects on the gut–adipose axis are manifested through multiple pathways that improve lipid metabolism: GLP-1 can activate the GLP-1 receptor (GLP-1R) in adipocytes, increase AMPK-PGC1α-UCP1 signaling activity, and induce adipose thermogenesis and lipolysis (94). Moreover, it can enhance intestinal barrier function by upregulating the activity of the PI3K/AKT/HIF-1α pathway, reducing circulating LPS levels, and indirectly alleviating WAT inflammation (95). Its receptor agonists, such as liraglutide, can also upregulate adiponectin by inhibiting the MKK4/JNK signaling pathway, improving adipose tissue-liver axis function, and reducing hepatic fat deposition (96).

**Figure 2:**
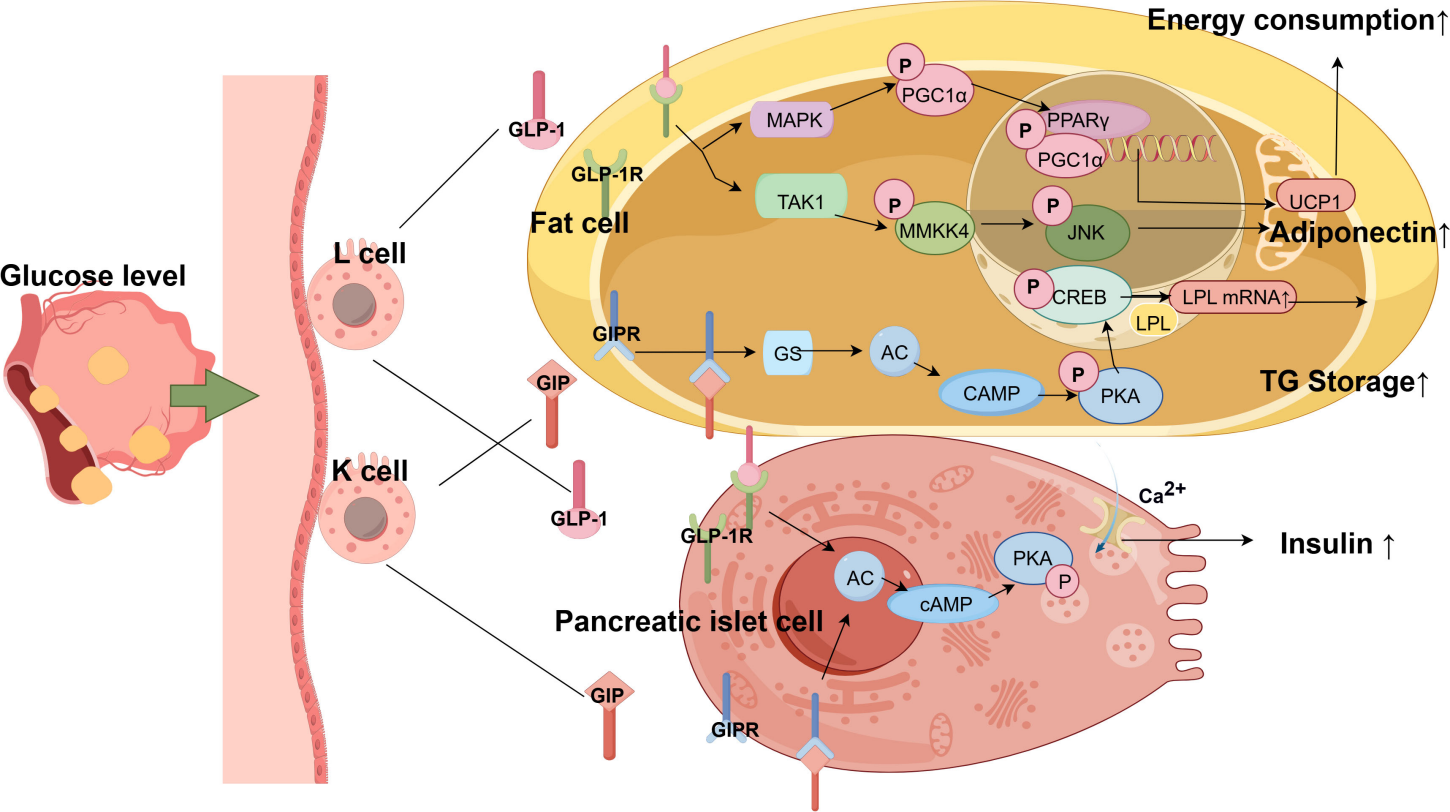
Mechanism of inulin in the dual-axis system. (GLP-1 secreted by intestinal L cells and GIP secreted by intestinal K cells bind to receptors on target cells, activating the Gs protein-adenylate cyclase-cAMP-PKA signaling pathway, thereby promoting insulin secretion from pancreatic β cells. Within adipocytes, GIP-1 enhances LPL-mediated triglyceride storage via this pathway. Furthermore, activation of GLP-1R in adipocytes triggers the TAK1-MKK4-JNK pathway to regulate adiponectin expression, while AMPK-mediated phosphorylation of PGC1α promotes PPARγ-dependent UCP1 expression and thermogenesis. AC, adenylate cyclase; AMPK, AMP-activated protein kinase; cAMP, cyclic adenosine monophosphate; Gs, G-protein stimulatory; JNK, c-Jun N-terminal kinase; LPL, lipoprotein lipase; MKK4, mitogen-activated protein kinase kinase 4; PGC1α, PPARγ coactivator-1α; PKA, protein kinase A; PPARγ, peroxisome proliferator-activated receptor gamma; TAK1, TGF-β-activated kinase 1; TG, triglyceride; UCP1, uncoupling protein 1.).

Comorbidity mechanism and therapeutic prospects: Although GLP-1 and GIP stimulate insulin secretion, they exhibit a “division of labor” in fat metabolism: GLP-1 (**Figure 2**) promotes energy expenditure and lipid breakdown, whereas GIP promotes the safe storage of lipids in adipose tissue. In pathological states, the responsiveness of both may be diminished; i.e., the “incretin effect” is reduced, collectively leading to islet functional decompensation and abnormal lipid metabolism distribution. This explains why dual GLP-1/GIP receptor agonists such as cotadutide, by synergistically activating both pathways, can not only significantly lower blood glucose but also more effectively reduce body weight and improve hepatic steatosis and inflammation, making them promising therapies for the comorbidity of T2DM, obesity, and MAFLD (97).

## 5 Therapeutic prospects

### 5.1 FMT and probiotics

Current strategies for managing metabolic syndrome focus primarily on weight loss, such as through increased physical activity and dietary changes. However, maintaining these habits in the long term is challenging. Thus, bariatric surgery has proven to be the most effective method for controlling obesity and thereby improving obesity-related comorbidities. Common surgical techniques include sleeve gastrectomy (SG) and Roux-en-Y gastric bypass (RYGB). SG is a restrictive procedure that removes most of the stomach. RYGB creates a small gastric pouch connected to the distal jejunum, resulting in both restrictive and malabsorptive effects. This approach can influence GLP-1 secretion, further improving glucose and lipid metabolism. Given the irreversible nature of these surgeries, fecal microbiota transplantation (FMT) has been explored in recent years (98). FMT involves transplanting microbiota from healthy donors via colonoscopy, enema, or specialized oral preparations to restore gut microbiota diversity and health. This microbiota-targeted approach is more acceptable for patients with metabolic syndrome and shows great potential for treating metabolic diseases.

However, FMT remains at an experimental stage for treating metabolic syndrome, similarly constrained by small sample sizes and limited intervention duration. Consequently, precision modulation of the gut microbiota through clearly defined probiotics—moving beyond broad-spectrum approaches—has emerged as a highly promising strategy. The selection criteria for such probiotics increasingly emphasize restoring specific functions impaired in metabolic syndrome. Examples include supplementing strains that produce SCFAs – such as certain Lactobacillus and Bifidobacterium species – to enhance intestinal barrier function and systemic metabolism. In a rat model, Liang et al. (99) confirmed that oral probiotics can modulate the composition of the gut microbiota, alter SCFAs levels, reduce body lipid deposition, and promote insulin release, demonstrating significant effects on reducing hepatic lipid deposition and blood glucose levels. *In vivo* animal studies revealed (100) that oral polysaccharide administration activates the SCFA/GPR43/GLP-1 pathway, thereby normalizing blood glucose levels in mice exhibiting glucose and lipid metabolism abnormalities induced by tacrolimus-induced disruption of gut butyrate-producing microbiota. Furthermore, mechanistic studies confirmed that administering specific Bifidobacterium strains can alleviate metabolic syndrome, and *Bifidobacterium animalis* ssp. *lactis GCL2505* can further reduce visceral fat and improve glucose tolerance (101). A recent Japanese cross-sectional study (102) revealed *Weizmannia* (formerly *Bacillus*) as a genus highly associated with metabolic syndrome. Oral administration of this bacterium was shown to affect mice liver fat accumulation and glucose tolerance by altering amino acid metabolism. Although related experiments are ongoing and targeted intestinally derived pharmaceuticals have not yet been developed, their potential role cannot be ignored. These approaches focusing on gut regulation—such as restoring microbial balance, blocking LPS leakage, and reshaping systemic metabolic homeostasis by breaking the vicious cycle—may become novel noninvasive strategies for intervening in MAFLD and T2DM in the future.

### 5.2 Receptor-targeted therapies

Rimonabant, initially approved in the EU in 2007 for improving lipid metabolism and insulin sensitivity, was later withdrawn from the market due to severe central nervous system side effects, including the worsening of pre-existing depression and suicidal tendencies, resulting from CB1 receptor blockade (103, 104). This case underscores the importance of precise receptor targeting in metabolic disease therapy. Research on CB1R, however, continues. For instance, INV-202, a peripherally restricted CB1R inhibitor currently in clinical trials, has not shown CNS-related side effects to date, indicating its potential as a novel therapeutic agent (105).

Owing to the effector role of incretins in the gut–adipose and gut–pancreatic axes, the safe and well-tolerated agents GLP-1 and GIP, beyond their use as glucose-lowering drugs, are now widely investigated for the treatment of MAFLD. Liraglutide (a long-acting GLP-1 analogue) and semaglutide (a GLP-1 receptor agonist) have been confirmed by clinical controlled trials to slow hepatic steatosis and non-alcoholic steatohepatitis, in addition to affecting insulin release (106, 107). Furthermore, a randomized controlled trial this year (108) demonstrated that treatment with the dual GLP-1/GIP receptor agonist pemvidutide significantly improved blood glucose levels, reduced liver fat content, improved liver inflammatory activity, and reduced body weight. These findings indicate that incretin-related agents may be effective for treating metabolic dysfunction-associated steatohepatitis and obesity.

### 5.3 Dietary intervention

Dietary intervention is a cornerstone strategy for managing MAFLD and T2DM and targeting the “gut–adipose/pancreatic axis”. Its core lies in reshaping the gut microbiota ecosystem by optimizing dietary structure, thereby positively regulating downstream metabolic signals. The Mediterranean diet (MD), which is rich in dietary fiber, polyunsaturated fatty acids, and polyphenols, is a prime example. Adherence to the MD is associated with reduced blood sugar and liver fat deposition rates in patients (109, 110). Mitsou et al. (111) reported that individuals with high MD scores had lower *Escherichia coli* counts, higher *Bifidobacterium*/*Escherichia coli* ratios, and higher acetate molar ratios than subjects with low adherence. De Filippis et al. (112) reported that among subjects consuming a seemingly Western diet, high consumption of plant-based foods consistent with the MD was associated with a beneficial microbiome-related metabolomic profile (higher abundance of *Prevotella* and certain fiber-degrading *Firmicutes* and higher SCFAs production). The elevated intestinal SCFAs levels in MD adherents are determined by high consumption of vegetables, fruits, and legumes, which are rich sources of complex and insoluble fibers, the primary substrates for microbial SCFAs production. Therefore, remodeling the gut microenvironment through diet can send beneficial metabolic regulation instructions simultaneously to both the gut–adipose axis and the gut–pancreatic axis, representing an effective nonpharmacological means to break the vicious cycle of glucose and lipid metabolism.

## 6 Discussion

In recent years, extensive research has investigated the gut–organ axes, which encompass various functional categories and exert broad influences on host behavior, metabolism, appetite, growth, reproduction, and immunity. Currently, numerous studies focus on revealing the role and mechanisms of gut-related dysfunction in metabolic diseases to explore new treatment strategies. The roles of adipose tissue and the pancreas in gut–organ axis crosstalk have gained widespread recognition and significant focus. In summary, this review moves beyond traditional cascade models to propose the new paradigm of “Common Messengers, Dual-Axis Branching” for MAFLD and T2DM comorbidity. This paradigm emphasizes that a limited set of core gut-derived messengers, by activating different downstream axes—namely, the gut–adipose axis and the gut–pancreatic islet axis—simultaneously yet distinctly disrupt lipid metabolism and glucose metabolism, respectively. This model not only explains the high frequency of comorbidity but also provides a revolutionary theoretical basis for developing therapies that simultaneously target multiple pathways. Although microbiome research is still in the development stage, numerous studies have confirmed associations between hormones and the gut microbiota. The perspective of centring on the gut and formulating preventive strategies aimed at reducing metabolic complications via the gut–systemic axes is gradually gaining prominence. Future work needs to further explore the mechanisms of cross-talk between other organ axes in metabolism-related diseases.
