# Correction: The gut–adipose/pancreas axis: a novel perspective on glycolipid metabolism dysregulation in MAFLD and T2DM pathogenesis

**DOI:** 10.3389/fendo.2025.1743993

**Published:** 2025-11-21

**Authors:** Jiahui Liang, Xingyu Chen, Yunhang Chu, Yan Leng

**Affiliations:** 1 College of Traditional Chinese Medicine, Changchun University of Traditional Chinese Medicine, Changchun, Jinlin, China; 2 Department of Liver, Spleen and Gastroenterology, Affiliated Hospital of Changchun University of Traditional Chinese Medicine, Changchun, Jilin, China

**Keywords:** metabolic-associated fatty liver disease, type 2 diabetes mellitus, gut microbiota, glucose and lipid metabolism, gut–adipose axis, gut–pancreatic axis

Affiliation 1 “College of Traditional Chinese Medicine, Changchun University of Traditional Chinese Medicine, Changchun, Jinlin, China” was erroneously given as “^1^Gastroenterology, Changchun University of Traditional Chinese Medicine, Changchun, Jilin, China”.

The order of authors in the author list of the published paper was erroneously given as:

“Jiahui Liang, Yunhang Chu, Xingyu Chen and Yan Leng”

The correct author list reads:

“Jiahui Liang, Xingyu Chen, Yunhang Chu and Yan Leng”

The original version of this article has been updated.

